# Supplementary material for: Transcriptional programming and T cell receptor repertoires distinguish human lung and lymph node memory T cells
Source: Commun Biol. 2019 Nov 13;2:411. doi: 10.1038/s42003-019-0657-2 (PMC6853923; doi:10.1038/s42003-019-0657-2)
Supplement: Supplementary file 1 — Supplementary Information [file 42003_2019_657_MOESM1_ESM.pdf]

Supplementary Figure 1. Flow cytometry and FACS gating strategy. Gating of populations from the LDLN of one donor (**a**). Gating of populations from the lung of the same donor (**b**).

a

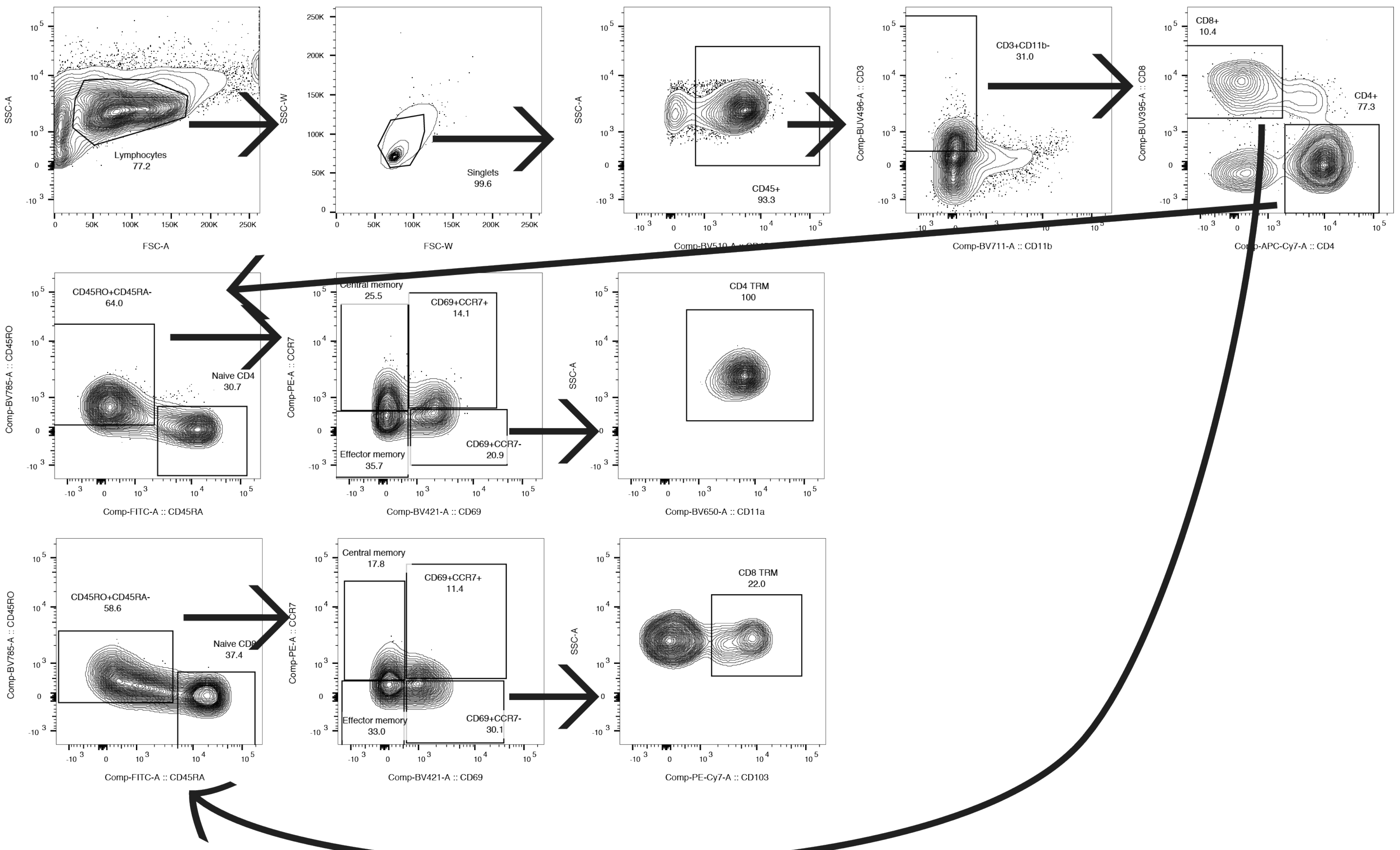

b

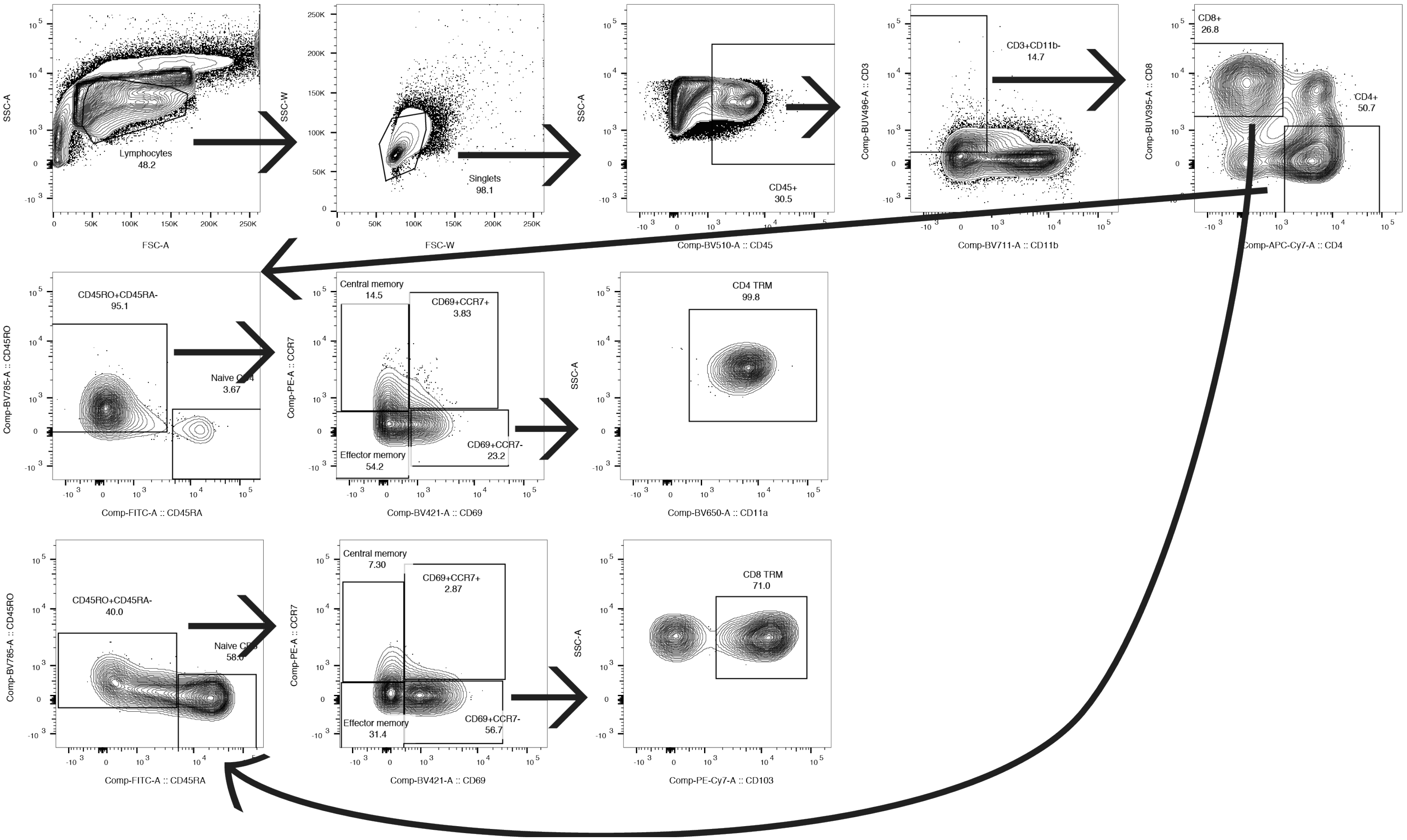

SSC-A
FSC-A
Lymphocytes
48.2
SSC-W
FSC-W
Singlets
98.1
Comp-BV510-A :: CD45
CD45+
30.5
Comp-BUV496-A :: CD3
Comp-BV711-A :: CD11b
CD3+CD11b-
14.7
Comp-BUV395-A :: CD8
Comp-APC-Cy7-A :: CD4
CD8+
26.8
CD4+
50.7
Comp-BV785-A :: CD45RO
Comp-FITC-A :: CD45RA
CD45RO+CD45RA-
95.1
3.67
Comp-PE-A :: CCR7
Comp-BV421-A :: CD69
Central memory
14.5
CD69+CCR7+
3.83
Effector memory
54.2
CD69+CCR7-
23.2
Comp-BV650-A :: CD11a
CD4 TRM
99.8
CD45RO+CD45RA-
40.0
Central memory
7.30
CD69+CCR7+
2.87
Effector memory
31.4
CD69+CCR7-
56.7
Comp-PE-Cy7-A :: CD103
CD8 TRM
71.0

Supplementary Figure 2. Boxplots of the frequency of T cell populations in paired lung and LDLN samples (n=11 for each boxplot). *P* value for paired t-test is indicated.

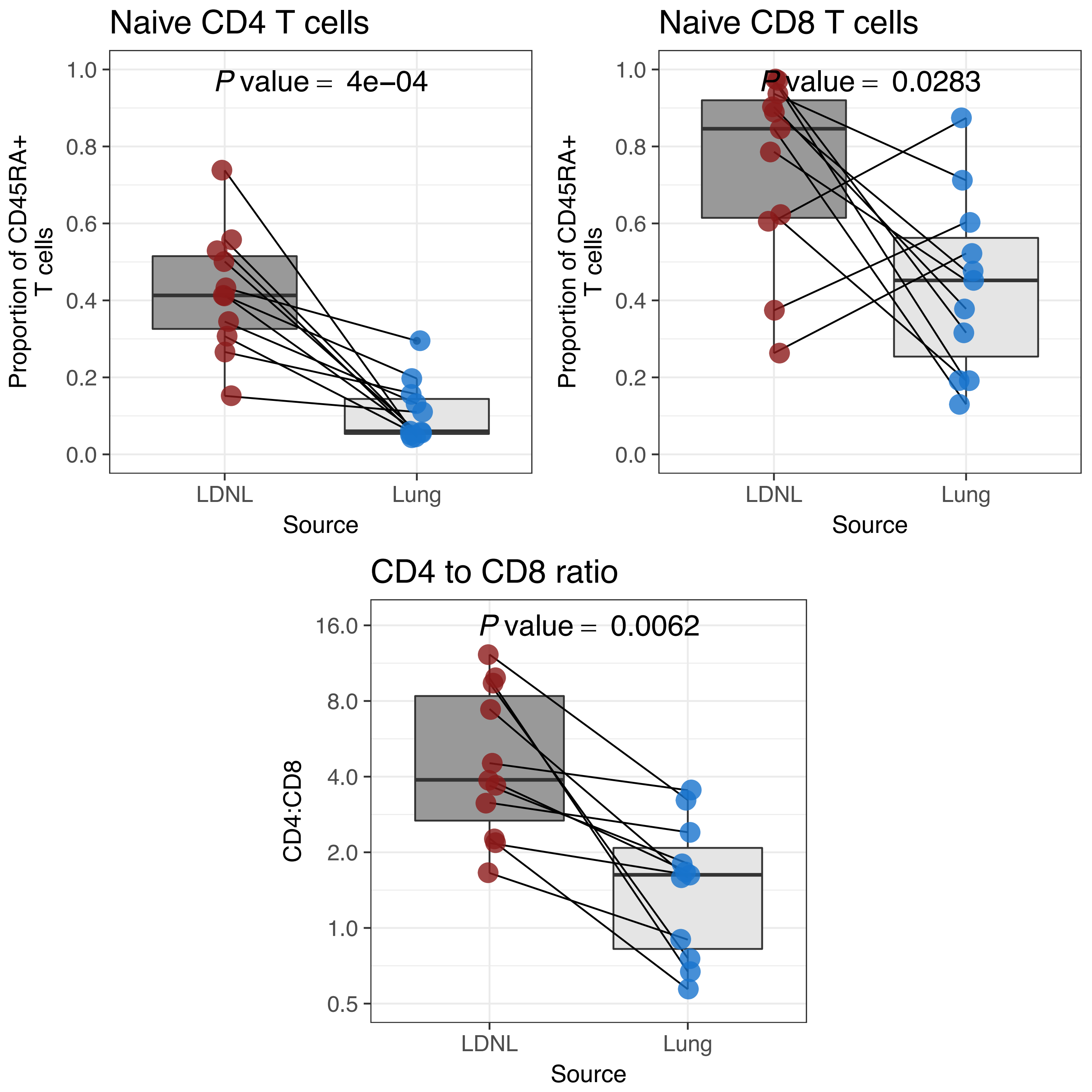

Supplementary Figure 3. T-distributed stochastic neighbor embedded plots colored by marker intensity.

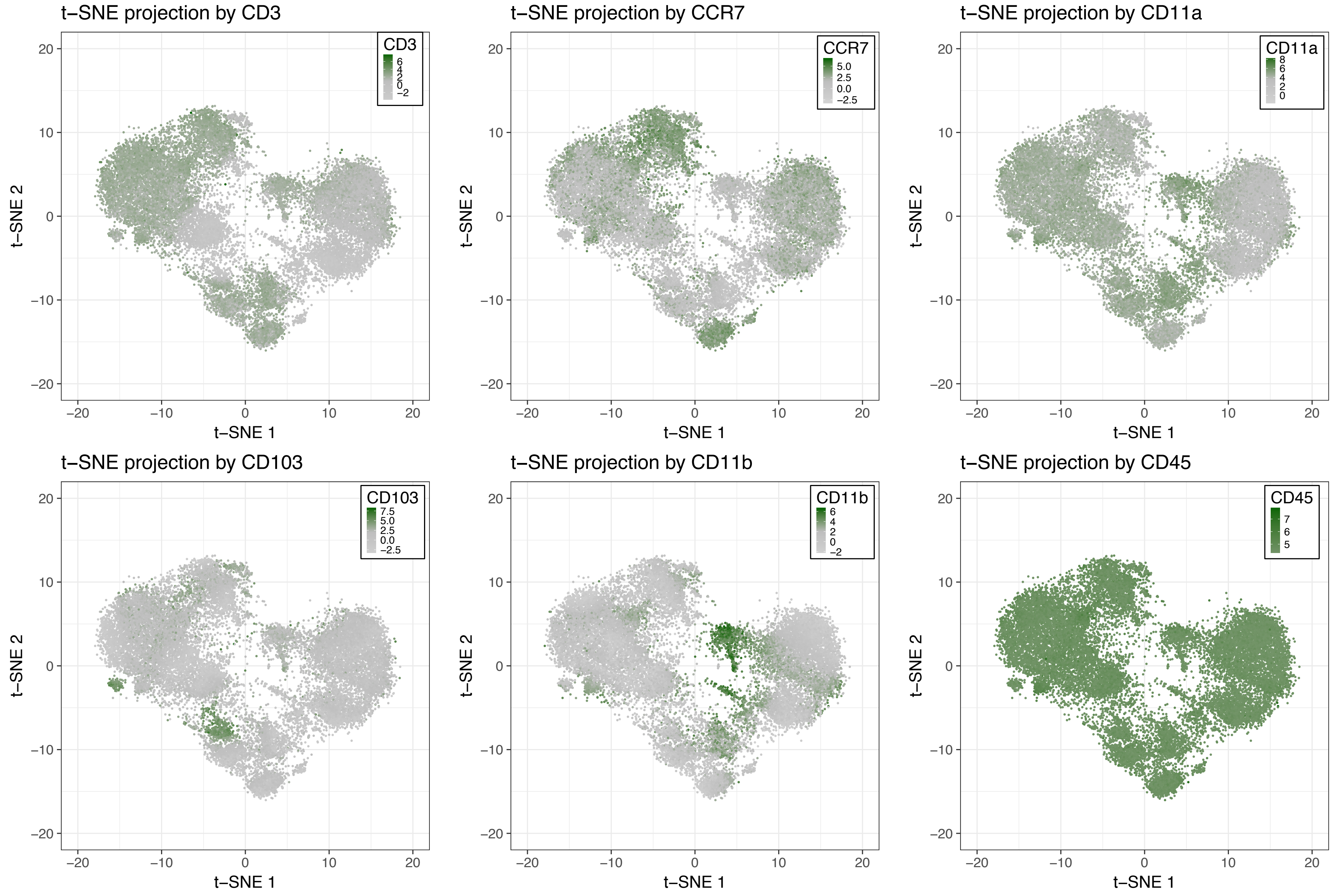

Supplementary Figure 4. Gene expression in counts per million (CPM) for CD4 and CD8B for each sample. The size of each point is proportional to the number of cells sorted for a given sample.

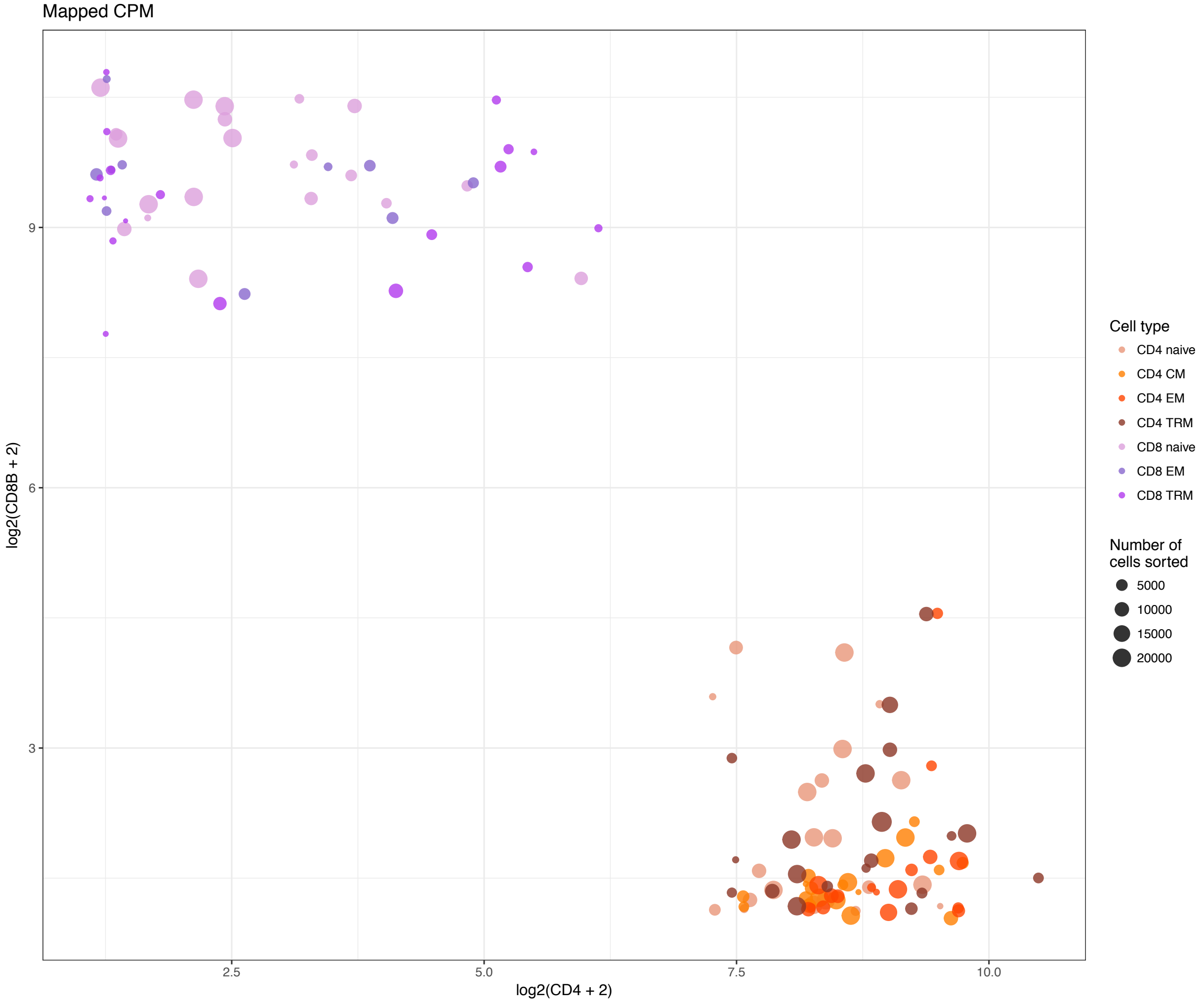

Supplementary Figure 5. Principal component analysis of RNA sequencing results after correcting for technical variables, age, gender and race.

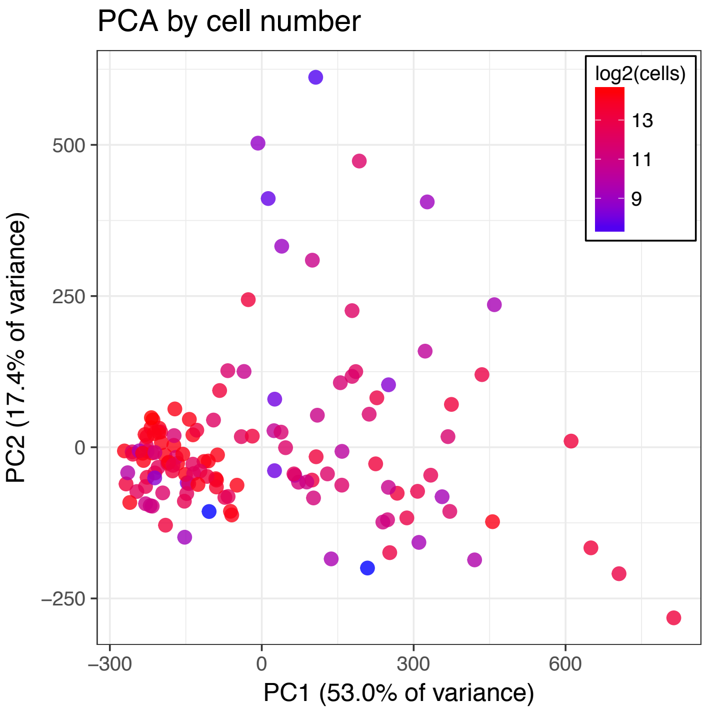

Supplementary Figure 6. Gene expression differences between lung and LDLN for CD4 EM.

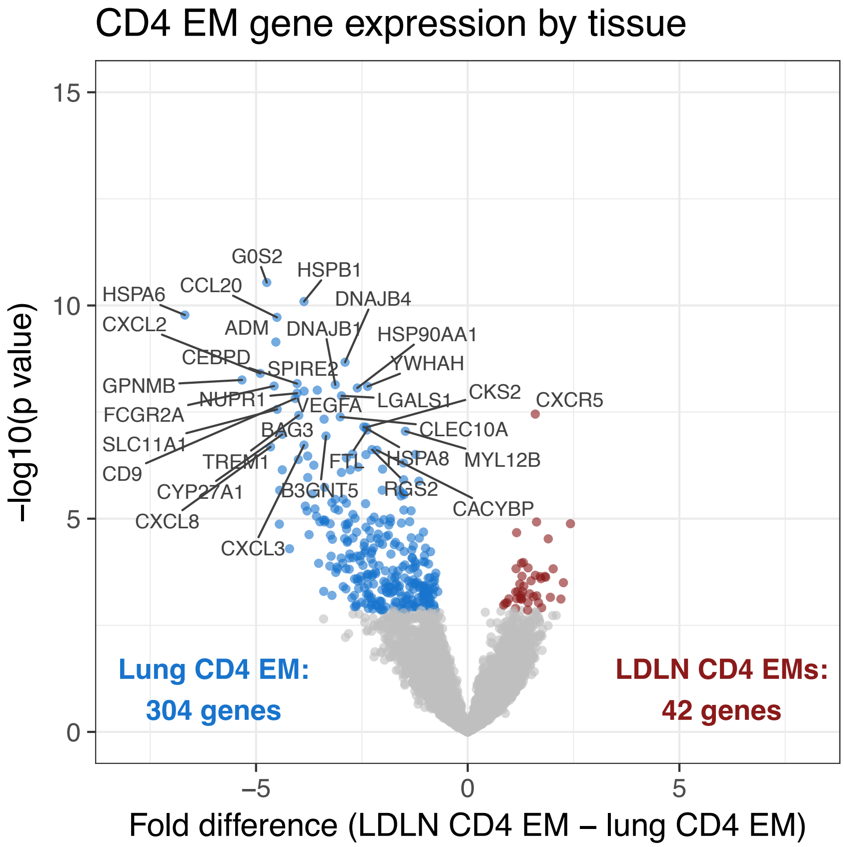

Supplementary Figure 7. Validation of gene expression differences by flow cytometry. Cell proportions for the markers shown in Figure 5. **a**, genes with higher expression in the lung. **b** genes with higher expression in the LDLN. **c** CD16 that was differentially expressed between all CD8 memory subsets.

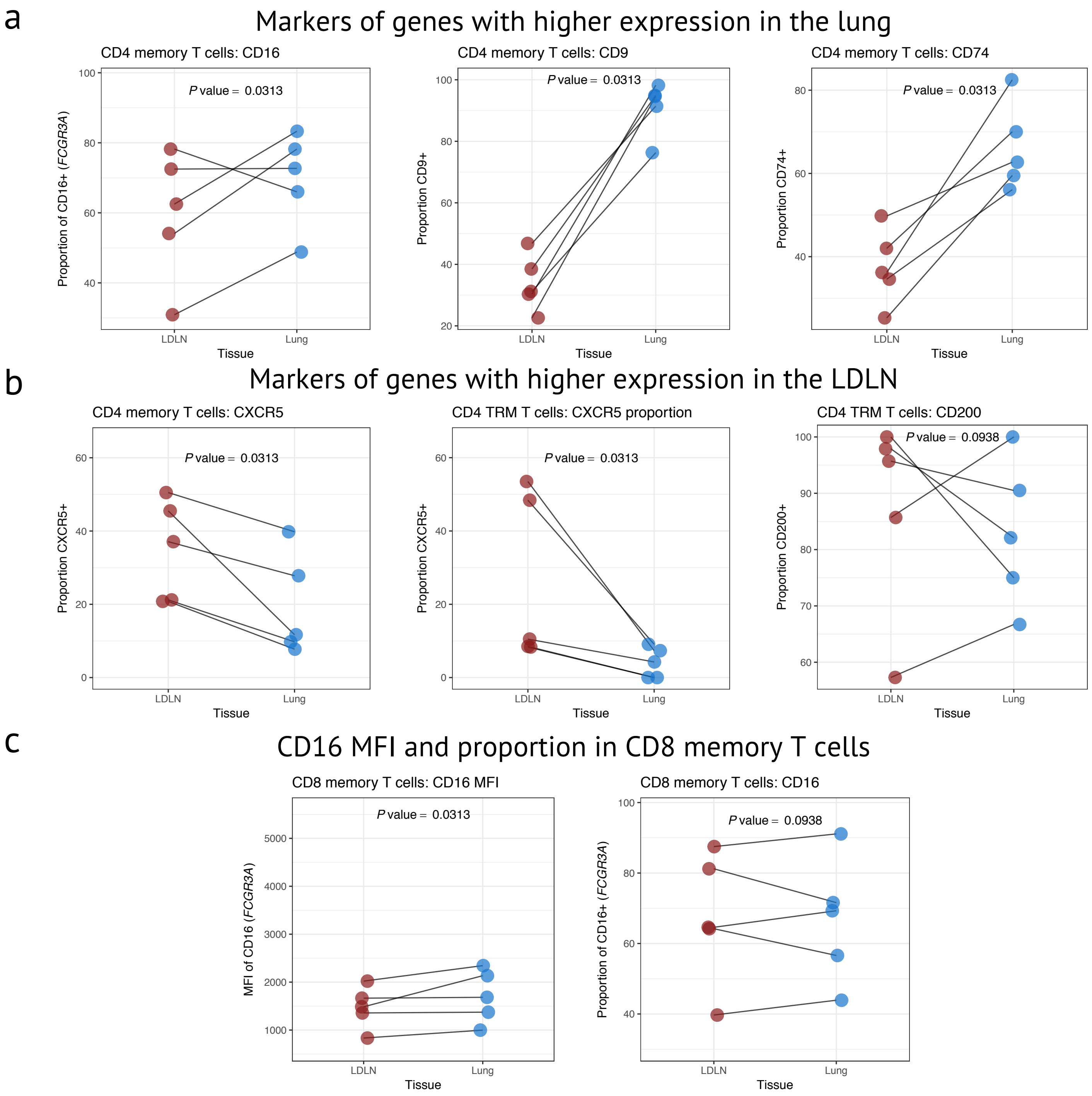

Supplementary Figure 8. Ingenuity Pathway Analysis networks. Networks from gene sets with higher expression in lung CD4 TRMs than LDLN CD4 TRMs, both with network scores of 54 (**a**). Networks generated from gene sets with higher expression in lung CD8 TRMs than and LDLN CD8 TRMs, all with network scores of 37 (**b**).

a

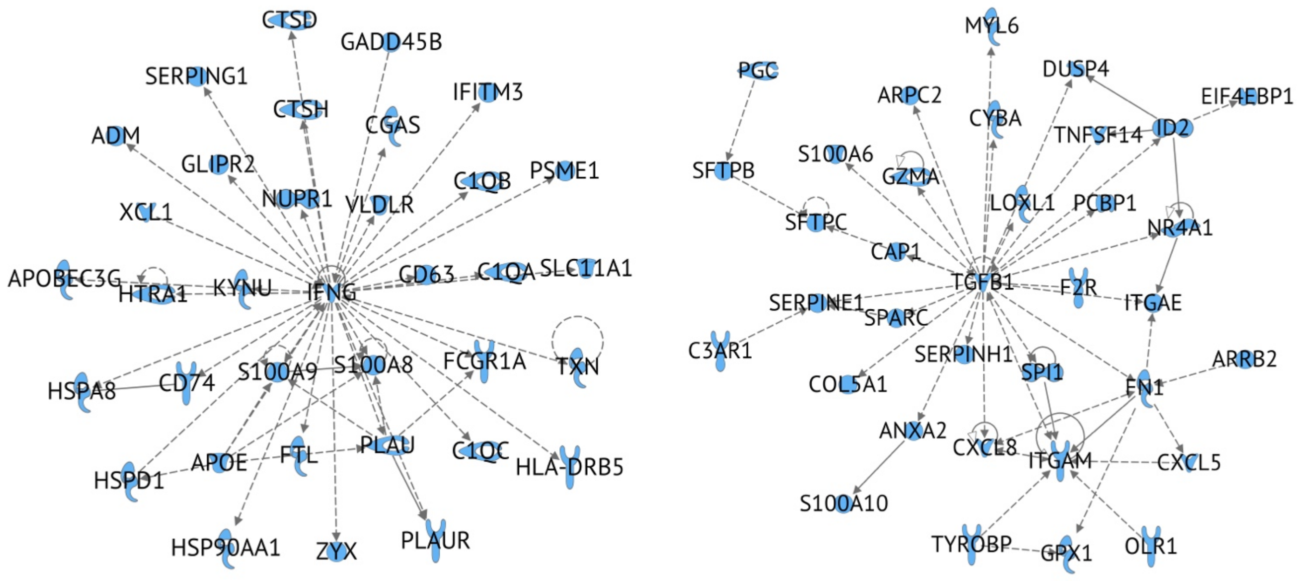

b

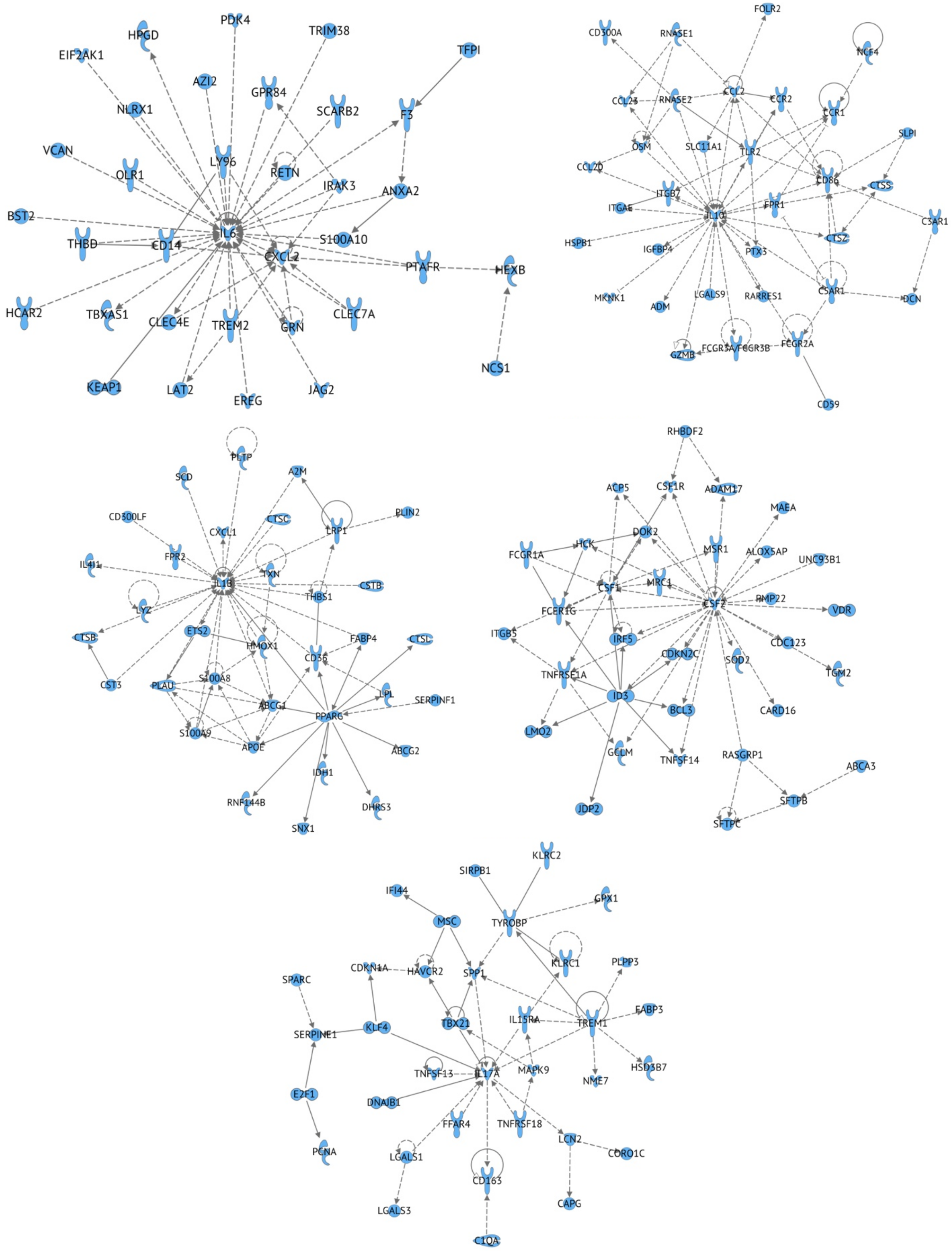
PDK4
HPGD
TRIM38
TFPI
EIF2AK1
AZI2
GPR84
SCARB2
NLRX1
F3
VCAN
LY96
RETN
OLR1
IRAK3
ANXA2
BST2
IL6
S100A10
THBD
CD14
CXCL2
PTAFR
HEXB
HCAR2
TBXAS1
CLEC4E
TREM2
GRN
CLEC7A
NCS1
KEAP1
LAT2
EREG
JAG2
FOLR2
CD300A
RNASE1
NCF4
CCL2
CCL23
RNASE2
CCR2
CCR1
OSM
SLC11A1
TLR2
SLPI
CCL20
CD86
ITGB7
CTSS
ITGAE
IL10
FPR1
C3AR1
HSPB1
IGFBP4
CTSZ
PTX3
MKNK1
ADM
LGALS9
RARRES1
C5AR1
DCN
GZMB
FCGR3A/FCGR3B
FCGR2A
CD59
PLTP
SCD
A2M
CD300LF
CXCL1
CTSC
LRP1
PLIN2
IL4I1
FPR2
TXN
IL1B
CSTB
THBS1
LYZ
CTSB
ETS2
HMOX1
FABP4
CTSL
CD36
CST3
PLAU
S100A8
LPL
ABCG1
SERPINF1
PPARG
S100A9
APOE
ABCG2
IDH1
RNF144B
DHRS3
SNX1
RHBDF2
ACP5
CSF1R
ADAM17
MAEA
DOK2
FCGR1A
HCK
MSR1
ALOX5AP
UNC93B1
CSF1
MRC1
FCER1G
CSF2
PMP22
VDR
ITGB5
IRF5
CDC123
CDKN2C
SOD2
TGM2
TNFRSF1A
ID3
BCL3
CARD16
LMO2
GCLM
TNFSF14
RASGRP1
ABCA3
JDP2
SFTPB
SFTPC
KLRC2
SIRPB1
IFI44
GPX1
MSC
TYROBP
KLRC1
PLPP3
CDKN1A
HAVCR2
SPP1
SPARC
FABP3
IL15RA
TREM1
TBX21
KLF4
SERPINE1
TNFSF13
IL17A
MAPK9
HSD3B7
NME7
E2F1
DNAJB1
FFAR4
TNFRSF18
LCN2
PCNA
LGALS1
CORO1C
CD163
LGALS3
CAPG
C1QA

Supplementary Figure 9. Rarefaction plot of TCR repertoires. Sample size (total number of TCRB CDR3 sequences) is plotted against diversity (number of clones). Each line indicates rarefaction with the dot indicating the measured number of sequences and diversity. Dotted line indicates extrapolated values for each sample and shading indicates the 95% confidence interval.

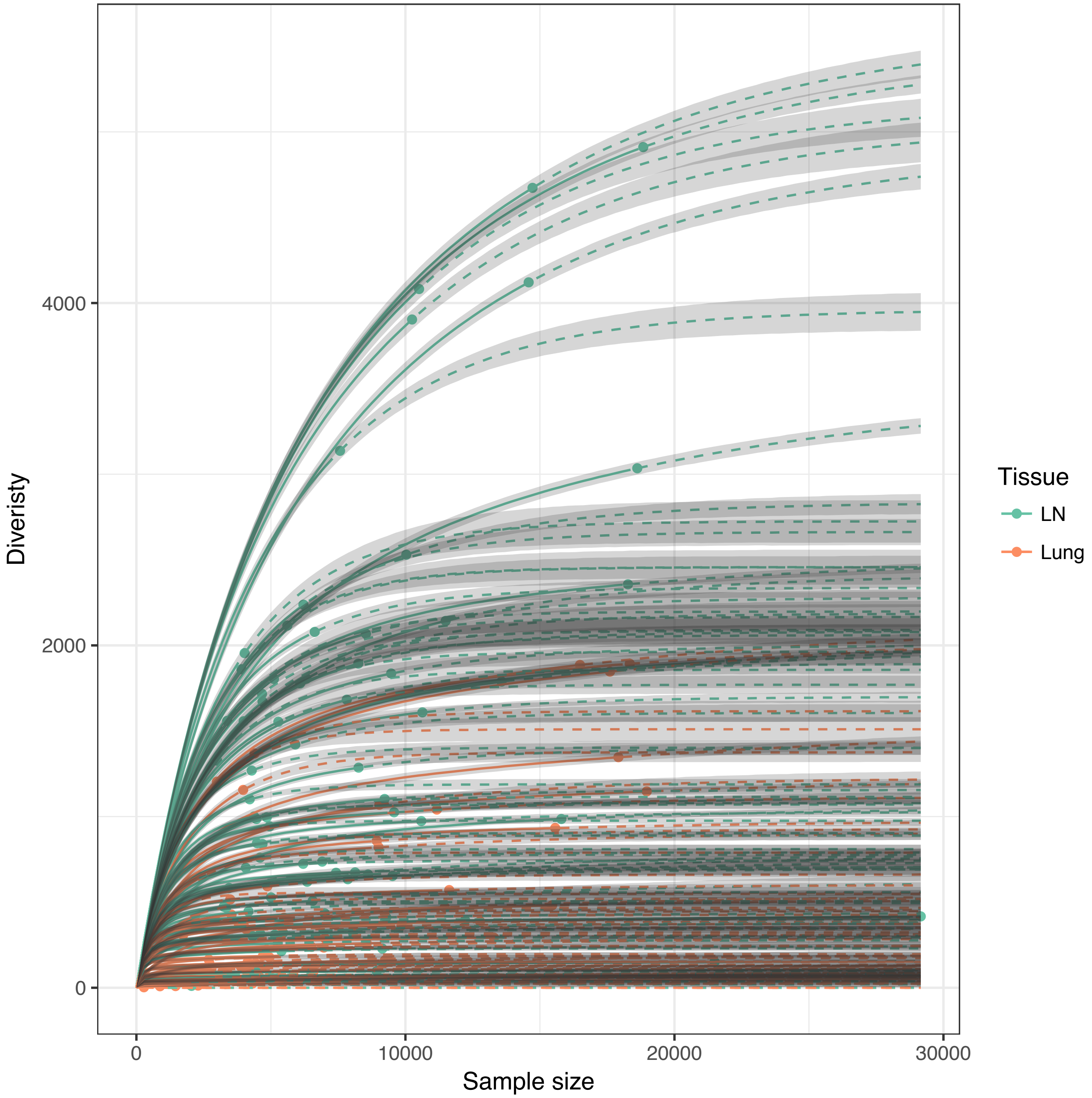

Supplementary Figure 10. T cell receptor beta clonal diversity of samples. Diversity was estimated using estimates from Chao(1).

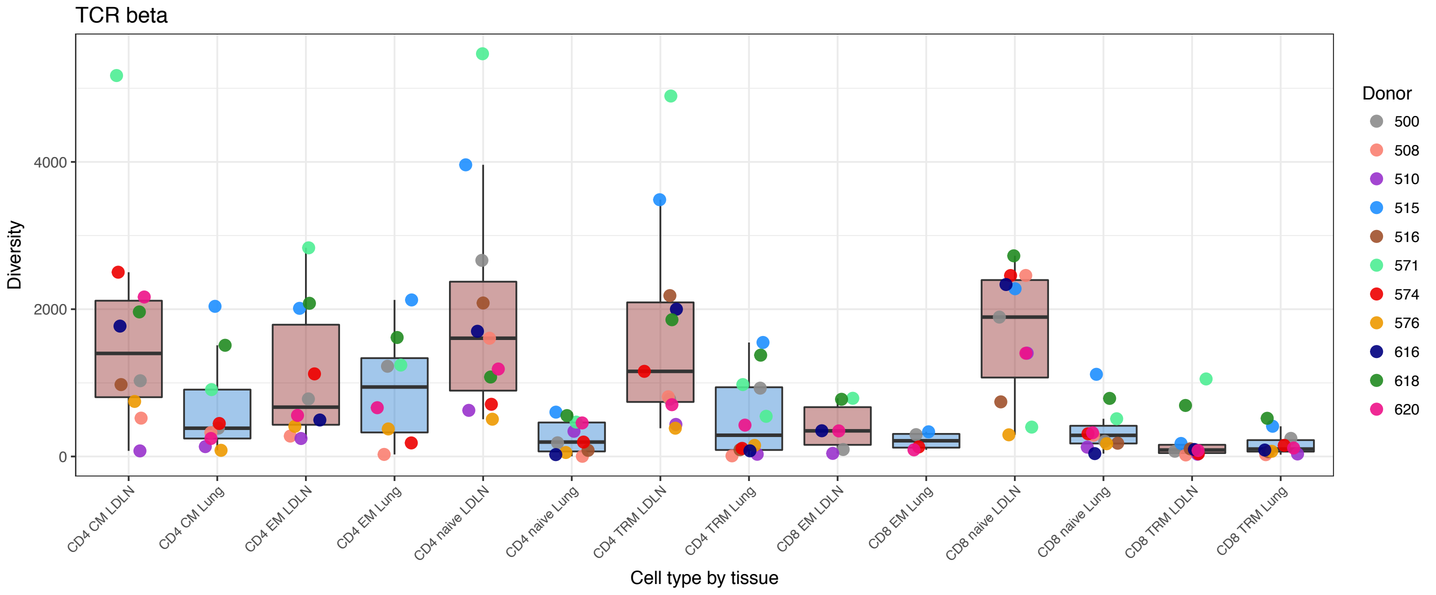

Supplementary Figure 11. Heatmap of clonal overlap between memory CD4 T cell subsets for donors not shown in Figure 7a.

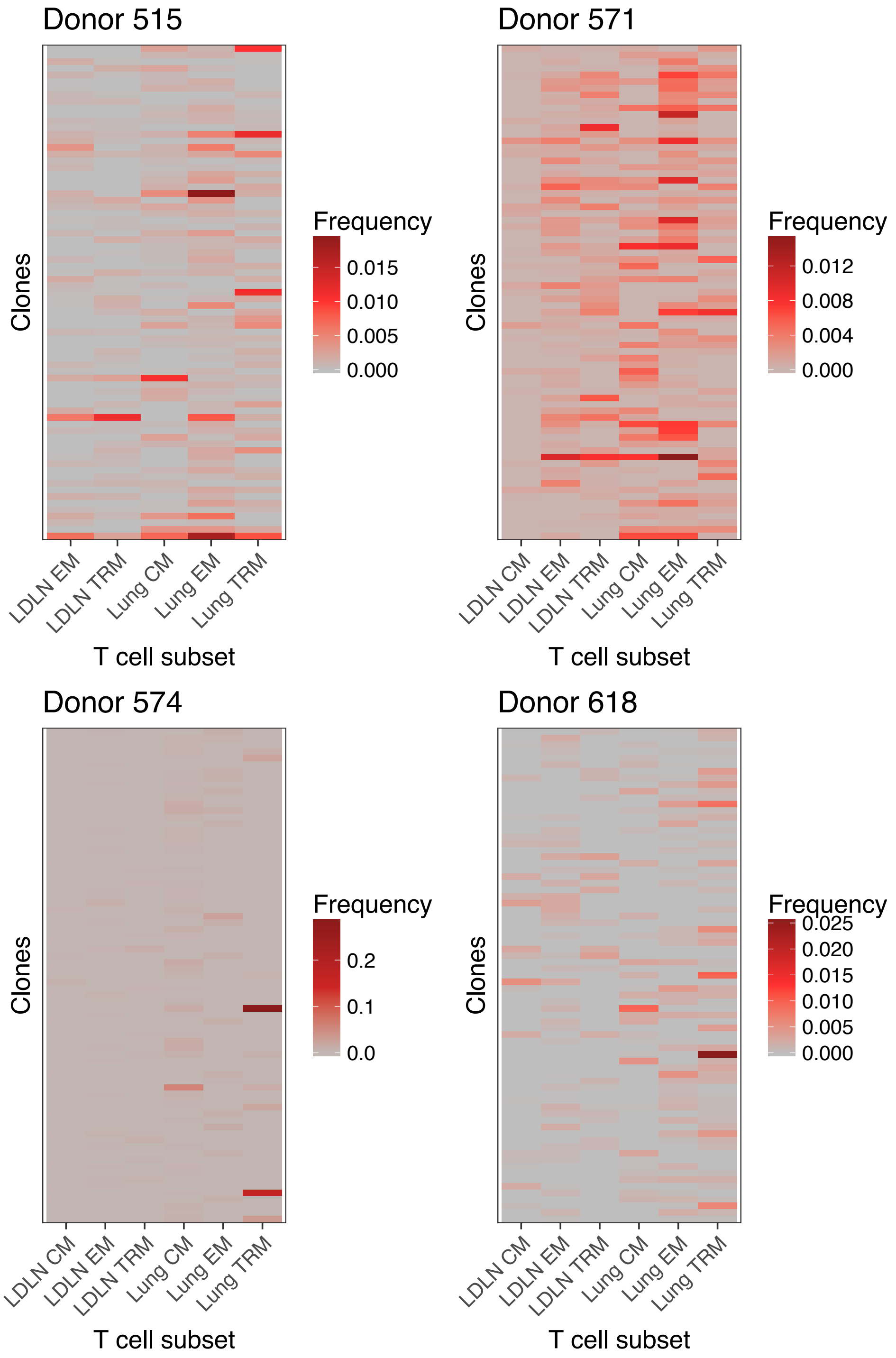

Supplementary Figure 12. Clustering of CD4 T cell subsets according to TCR repertoire overlap for donors not shown in Figure 7b.

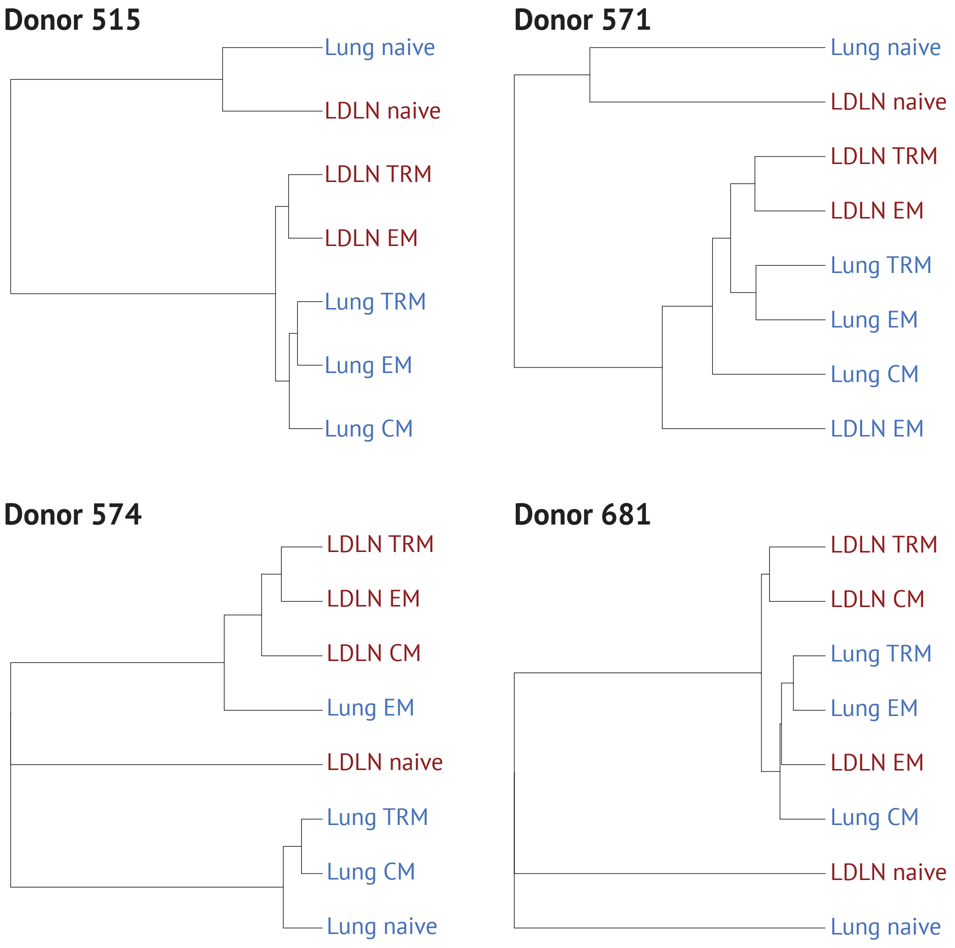

Supplementary Figure 13. Multidimensional scaling of TCR repertoires from CD4 and CD8 subsets colored by donor (top) and tissue (bottom).

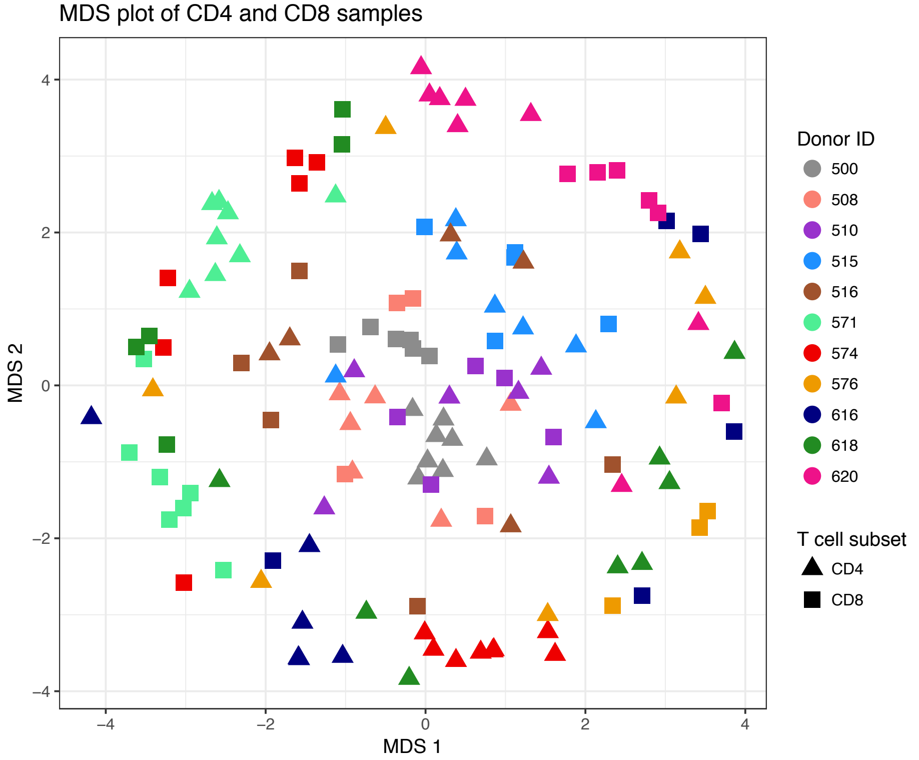

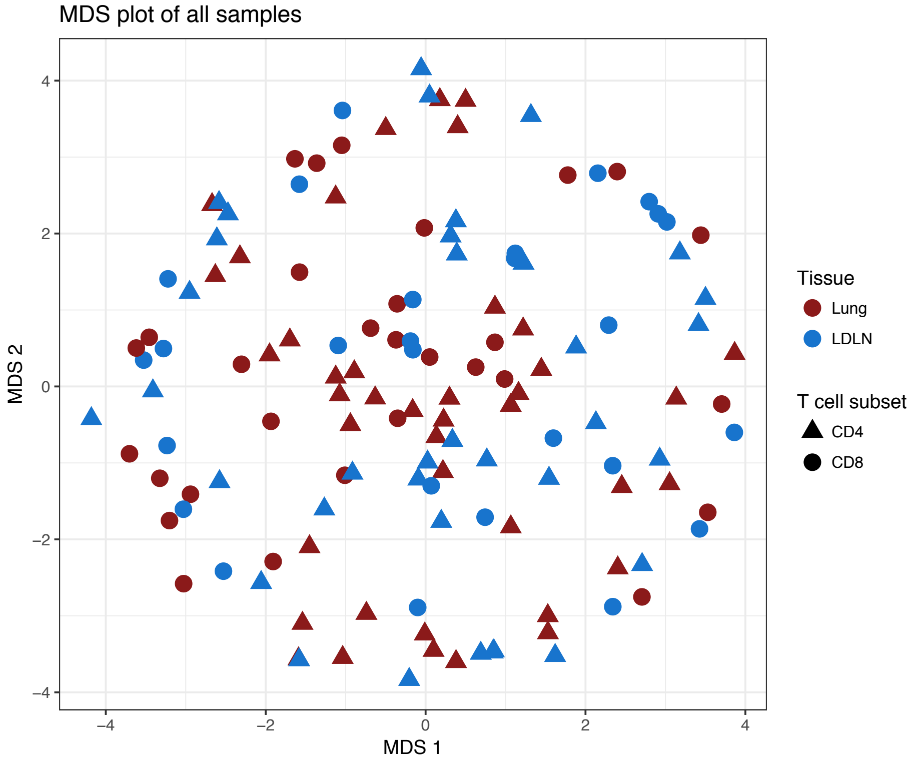

Supplementary Figure 14. Riverplots of TCR beta V and J gene usage. Each line connects a V gene segment with a J gene segment, and the width is proportional to the number of different clones with a given V-J recombination in a sample. V and J gene segments are ordered by nomenclature. Lines are colored by V-J gene combination and are identical across plots. Plots are shown for CD4 populations sorted from the lung and LDLN from donors 571 (**a**) and 618 (**b**).

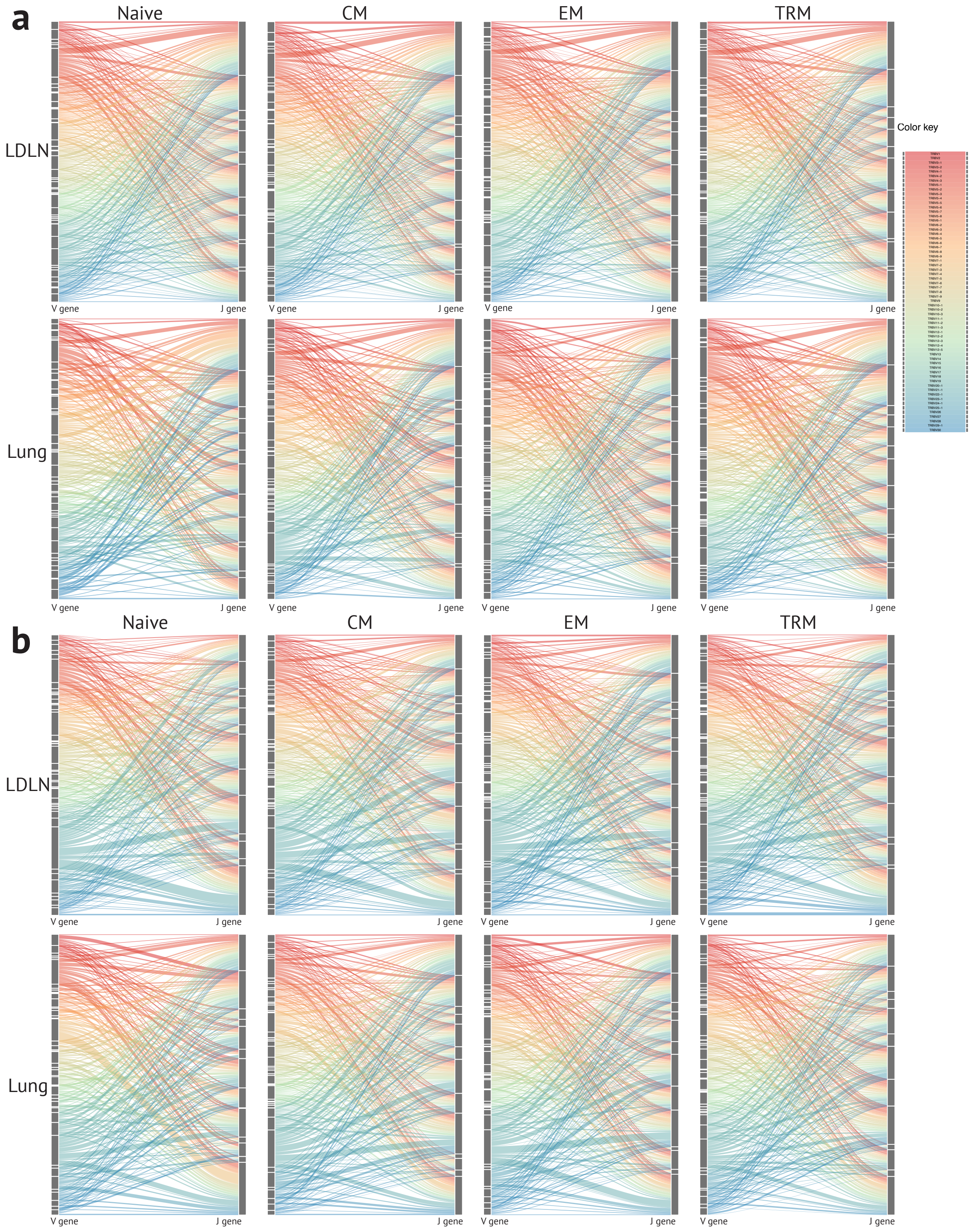

Supplementary Figure 15. V-J gene usage across CD4 T cell subsets from two donors, 500 and 620. Top row are plots for subsets from the lung and second are for subsets from the LDLN for 500. Third and fourth row are for lung and LDLN subsets from donor 620, respectively.

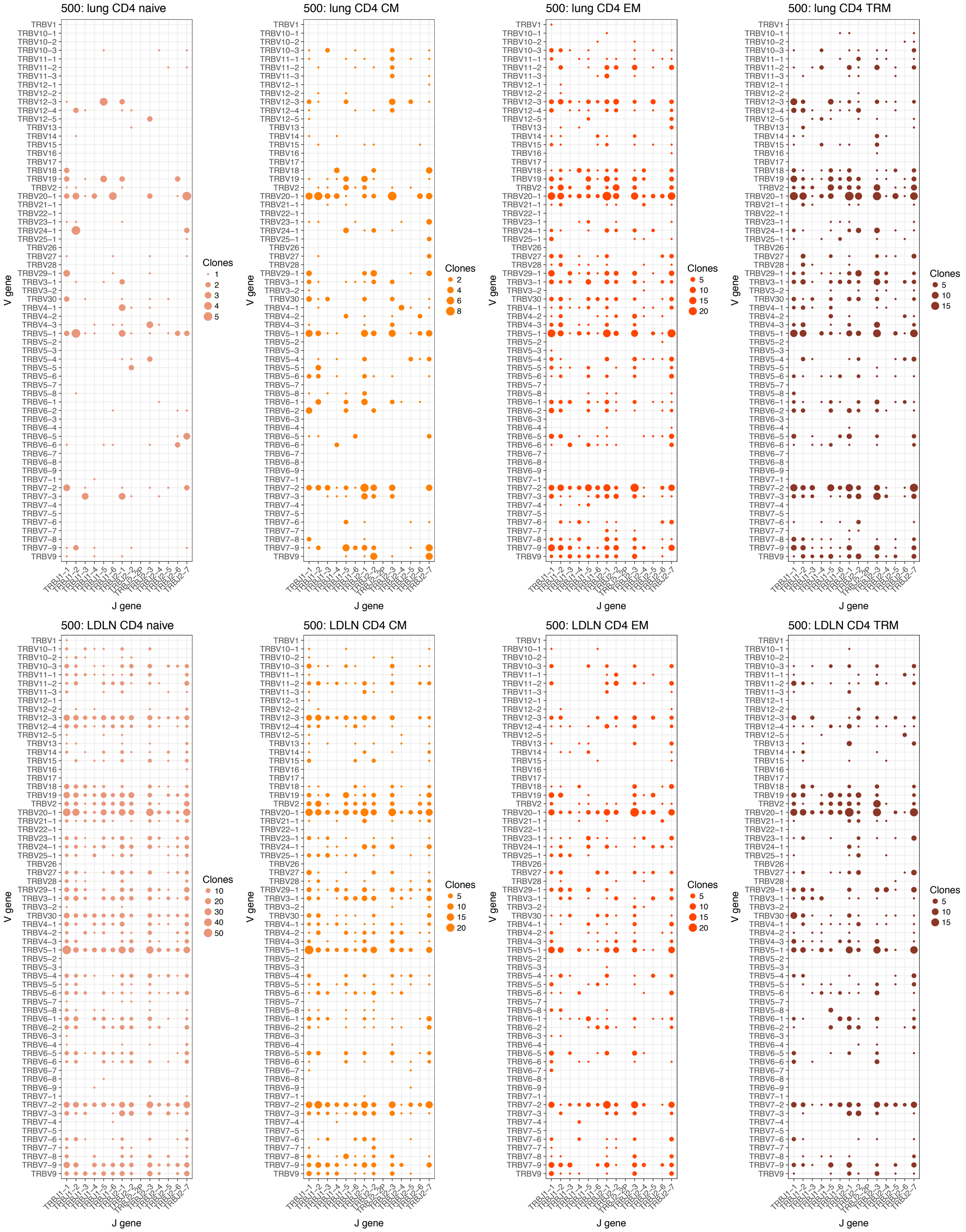

Supplementary Figure 15 continued.

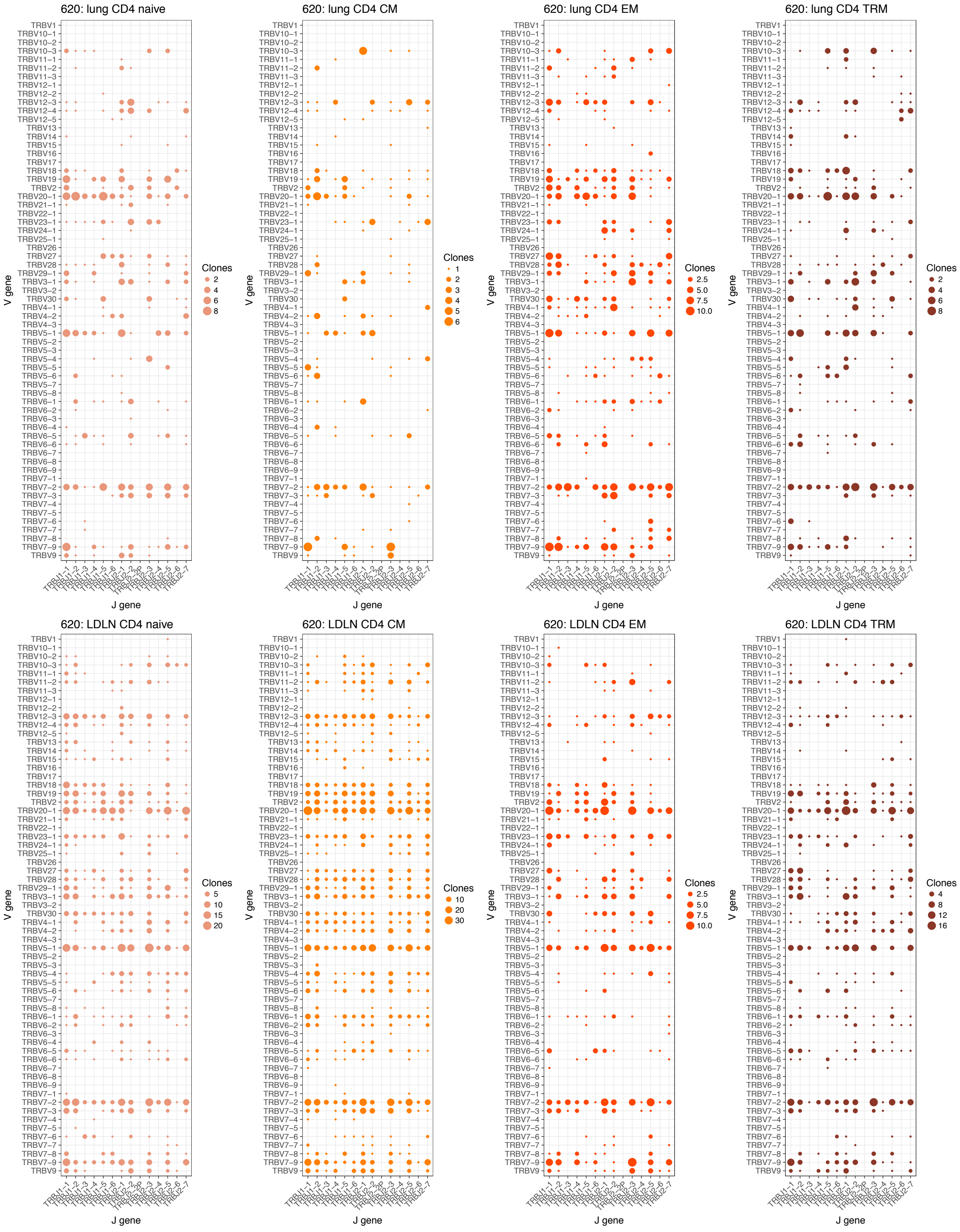

Supplementary Figure 16. V-J gene usage for all CD4 and CD8 T cells. Clones were pooled for all donors. **a** V-J gene usage for all CD4 T cell samples and separated by tissue. **b** V-J gene usage for all CD8 T cell samples and separated by tissue. The size of the dot is proportional to the number of clones.

a

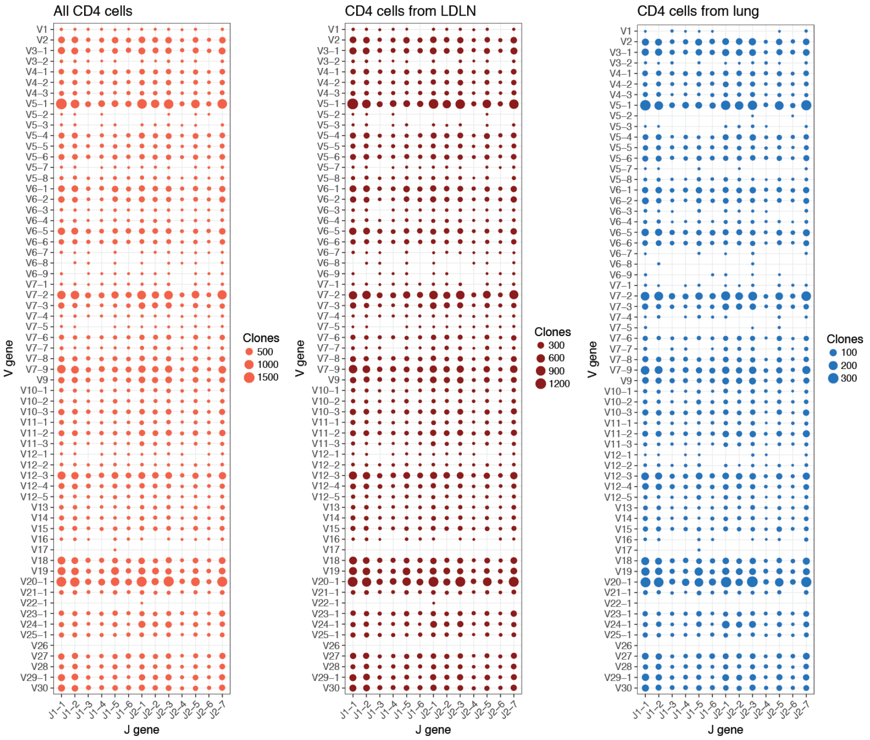

b

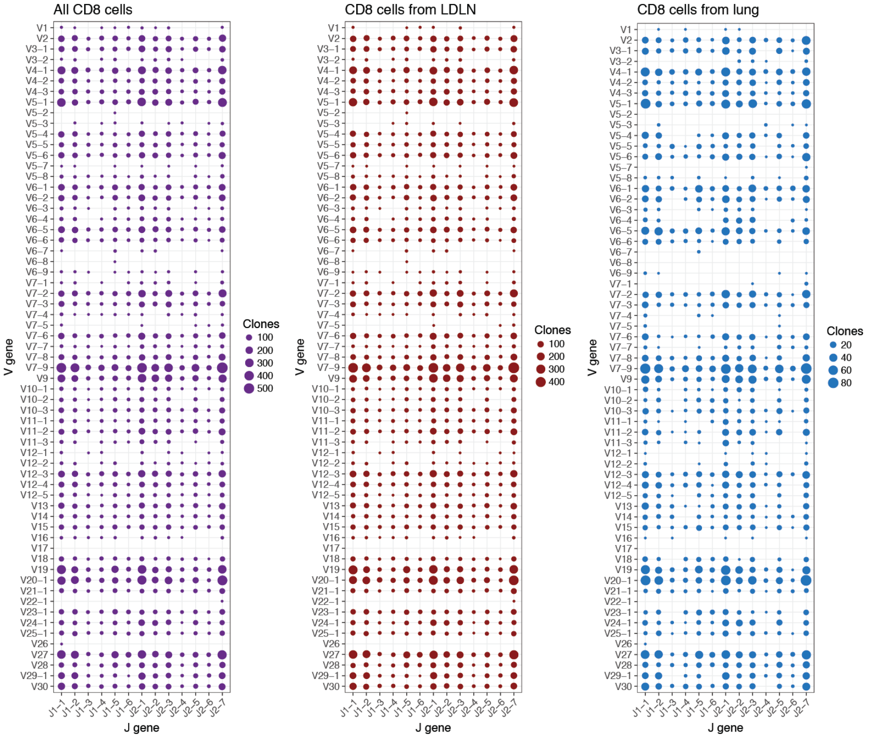

Supplementary Table 1. Antibody panel used for flow cytometry and FACS.

Marker	Fluorochrome	Manufacture	Clone	Volume per stain (µL per 1 million cells in 100 µL)
**Antibodies used for cell sorting and initial phenotyping**				
**CCR7**	PE	Biolegend	G043H7	5
**CD3**	BUV496	BD	UCHT1	5
**CD4**	APC-Cy7	Biolegend	RPA-T4	5
**CD8**	BUV395	BD	RPA-T8	5
**CD11a**	BV650	BD	HI111	5
**CD11b**	BV711	Biolegend	M1/70	2
**CD45RA**	FITC	BD	HI100	5
**CD45RO**	BV786	BD	UCHL1	5
**CD69**	BV421	Biolegend	FN50	5
**CD103**	PE-Cy7	Biolegend	Ber-ACT8	5
**CD169**	APC	Biolegend	7-239	5
**Additional antibodies used for validation of gene expression results**				
**CD45RA**	BUV737	BD	HI100	5
**CD185**	APC	Biolegend	J252D4	5
**CD16**	BV605	Biolegend	3G8	5
**CD74**	APC	Biolegend	LN2	5
**CD9**	FITC	Biolegend	HI9a	5
**CD200**	BV605	Biolegend	OX-104	5

Supplementary Table 2. Cell phenotypes.

CD subset	Cell type	Phenotype
CD4	Naïve	CD45+CD3+CD11b-CD4+CD8-RA+RO-
	CM	CD45+CD3+CD11b-CD4+CD8-RA-RO+CD69-CCR7+
	EM	CD45+CD3+CD11b-CD4+CD8-RA-RO+CD69-CCR7-
	TRM	CD45+CD3+CD11b-CD4+CD8-RA-RO+CD69+CCR7-CD11a+
CD8	Naïve	CD45+CD3+CD11b-CD4-CD8+RA+RO-
	CM	CD45+CD3+CD11b-CD4-CD8+RA-RO+CD69-CCR7+
	EM	CD45+CD3+CD11b-CD4-CD8+RA-RO+CD69-CCR7-
	TRM	CD45+CD3+CD11b-CD4-CD8+RA-RO+CD69+CCR7-CD103+

Supplementary References

1. Chao A (1987) Estimating the population size for capture-recapture data with unequal catchability. *Biometrics* 43(4):783-791.
