# Supplementary material for: Transcriptional programming and T cell receptor repertoires distinguish human lung and lymph node memory T cells
Source: Commun Biol. 2019 Nov 13;2:411. doi: 10.1038/s42003-019-0657-2 (PMC6853923; doi:10.1038/s42003-019-0657-2)
Supplement: Supplementary file 2 — Description of Supplementary Files [file 42003_2019_657_MOESM2_ESM.pdf]

Description of Additional Supplementary files

Supplementary Data 1: Samples and cell numbers sorted.
Supplementary Data 2: Genes differentially expressed between tissues for CD4 TRM.
Supplementary Data 3: Genes differentially expressed between tissues for CD4 EM.
Supplementary Data 4: Genes differentially expressed between tissues for CD4 CM.
Supplementary Data 5: Genes differentially expressed between tissues for CD8 TRM.
Supplementary Data 6: Genes differentially expressed between tissues for CD8 EM.
Supplementary Data 7: GO for genes differentially expressed by tissue for all CD4 memory subsets.
Supplementary Data 8: GO for genes differentially expressed by tissue for all CD8 memory subsets.
Supplementary Data 9: GO for genes differentially expressed by tissue in both CD4 TRM and CD8 TRM.
Supplementary Data 10: Pathway analysis of CD4 TRM.
Supplementary Data 11: Pathway analysis of CD8 TRM.
Supplementary Data 12: IPA results for genes with higher expression in lung TRM.
Supplementary Data 13: GO for genes differentially expressed by tissue for CD4 TRM but not other memory CD4 subsets.
Supplementary Data 14: GO for genes differentially expressed by tissue for CD8 TRM but not other memory CD8 subsets.
