# Supplementary material for: Transcriptional programming and T cell receptor repertoires distinguish human lung and lymph node memory T cells
Source: Commun Biol. 2019 Nov 13;2:411. doi: 10.1038/s42003-019-0657-2 (PMC6853923; doi:10.1038/s42003-019-0657-2)
Supplement: Supplementary file 4 — Reporting Summary [file 42003_2019_657_MOESM4_ESM.pdf]

nature research

Corresponding author(s): Schoettler, N

Last updated by author(s): Sep 27, 2019

Reporting Summary

Nature Research wishes to improve the reproducibility of the work that we publish. This form provides structure for consistency and transparency in reporting. For further information on Nature Research policies, see Authors & Referees and the Editorial Policy Checklist.

Statistics

For all statistical analyses, confirm that the following items are present in the figure legend, table legend, main text, or Methods section.

n/a	Confirmed	
☐	☒	The exact sample size (n) for each experimental group/condition, given as a discrete number and unit of measurement
☐	☒	A statement on whether measurements were taken from distinct samples or whether the same sample was measured repeatedly
☐	☒	The statistical test(s) used AND whether they are one- or two-sided<br>*Only common tests should be described solely by name; describe more complex techniques in the Methods section.*
☐	☒	A description of all covariates tested
☐	☒	A description of any assumptions or corrections, such as tests of normality and adjustment for multiple comparisons
☐	☒	A full description of the statistical parameters including central tendency (e.g. means) or other basic estimates (e.g. regression coefficient) AND variation (e.g. standard deviation) or associated estimates of uncertainty (e.g. confidence intervals)
☐	☒	For null hypothesis testing, the test statistic (e.g. F, t, r) with confidence intervals, effect sizes, degrees of freedom and P value noted<br>*Give P values as exact values whenever suitable.*
☒	☐	For Bayesian analysis, information on the choice of priors and Markov chain Monte Carlo settings
☐	☒	For hierarchical and complex designs, identification of the appropriate level for tests and full reporting of outcomes
☒	☐	Estimates of effect sizes (e.g. Cohen's d, Pearson's r), indicating how they were calculated

Our web collection on statistics for biologists contains articles on many of the points above.

Software and code

Policy information about availability of computer code

Data collection	Included in manuscript
Data analysis	Included in manuscript

For manuscripts utilizing custom algorithms or software that are central to the research but not yet described in published literature, software must be made available to editors/reviewers. We strongly encourage code deposition in a community repository (e.g. GitHub). See the Nature Research guidelines for submitting code & software for further information.

Data

Policy information about availability of data

All manuscripts must include a data availability statement. This statement should provide the following information, where applicable:

- Accession codes, unique identifiers, or web links for publicly available datasets
- A list of figures that have associated raw data
- A description of any restrictions on data availability

Raw RNA sequencing data and processed data are deposited in GEO with accession number GES137967

Field-specific reporting

Please select the one below that is the best fit for your research. If you are not sure, read the appropriate sections before making your selection.

☒ Life sciences ☐ Behavioural & social sciences ☐ Ecological, evolutionary & environmental sciences

For a reference copy of the document with all sections, see nature.com/documents/nr-reporting-summary-flat.pdf

Life sciences study design

All studies must disclose on these points even when the disclosure is negative.

Sample size	No sample size calculation was performed. The number of samples was based on availability of adequate quality samples.
Data exclusions	Certain analyses were not performed because of an insufficient number of samples (e.g. gene expression comparisons of lung and LDLN CD8 CM T cell subsets), and these are indicated in the text and methods.
Replication	Gene expression patterns in lung CD4 TRMs and lung CD8 TRMs has been replicated in separate experiments, using an alternate gating strategy for cell sorting.
Randomization	This is described in the manuscript. Briefly, donors could not be randomized, but at the time of sorting, cells from at least two donors were sorted on the same day.
Blinding	Blinding was not performed.

Reporting for specific materials, systems and methods

We require information from authors about some types of materials, experimental systems and methods used in many studies. Here, indicate whether each material, system or method listed is relevant to your study. If you are not sure if a list item applies to your research, read the appropriate section before selecting a response.

Materials & experimental systems

n/a	Involved in the study	
☐	☒	Antibodies
☒	☐	Eukaryotic cell lines
☒	☐	Palaeontology
☒	☐	Animals and other organisms
☐	☒	Human research participants
☒	☐	Clinical data

Methods

n/a	Involved in the study	
☒	☐	ChIP-seq
☐	☒	Flow cytometry
☒	☐	MRI-based neuroimaging

Antibodies

Antibodies used

Provided in Supplemental Table 1 and below.

Validation

All antibodies used are commercially available from BioLegend or BD Biosciences.
CCR7/G043H7/PE/Biolegend/https://www.biolegend.com/de-at/products/pe-anti-human-cd197-ccr7-antibody-7498
CD3/UCHT1/BUV496/BD Biosciences/http://www.bdbiosciences.com/us/reagents/research/antibodies-buffers/immunology-reagents/anti-human-antibodies/cell-surface-antigens/buv496-mouse-anti-human-cd3-ucht1-also-known-as-ucht-1-ucht-1/p/564809
CD4/RPA-T4/APC-Cy7/Biolegend/https://www.biolegend.com/en-us/products/apc-cyanine7-anti-human-cd4-antibody-1933
CD8/RPA-T8/BUV395/BD Biosciences/http://www.bdbiosciences.com/us/reagents/research/antibodies-buffers/immunology-reagents/anti-human-antibodies/cell-surface-antigens/buv395-mouse-anti-human-cd8-rpa-t8/p/563795
CD11a/HI111/BV650/BD Biosciences/http://www.bdbiosciences.com/us/applications/research/t-cell-immunology/regulatory-t-cells/surface-markers/human/bv650-mouse-anti-human-cd11a-hi111/p/563934
CD11b/M1/70/BV711/Biolegend/https://www.biolegend.com/fr-fr/products/brilliant-violet-711-anti-mouse-human-cd11b-antibody-7927
CD45RA/HI100/FITC/BD Biosciences/http://www.bdbiosciences.com/us/applications/research/t-cell-immunology/regulatory-t-cells/surface-markers/human/fitc-mouse-anti-human-cd45ra-hi100/p/555488
CD45RO/UCHL1/BV786/BD Biosciences/https://www.bdbiosciences.com/us/reagents/research/antibodies-buffers/immunology-reagents/anti-human-antibodies/cell-surface-antigens/bv786-mouse-anti-human-cd45ro-uchl1/p/564290
CD69/FN50/BV421/Biolegend/https://www.biolegend.com/en-us/products/brilliant-violet-421-anti-human-cd69-antibody-7141
CD103/Ber-ACT8/PE-Cy7/Biolegend/https://www.biolegend.com/fr-fr/products/pe-cy7-anti-human-cd103-integrin-alphae-antibody-8206
CD169/7-239/APC/Biolegend/https://www.biolegend.com/en-us/products/apc-anti-human-cd169-sialoadhesin--siglec-1-antibody-7772

All antibodies were used at the manufacture-recommended concentrations.

Human research participants

Policy information about studies involving human research participants

Population characteristics

This is reported for every donor in this study in the supplement.

Recruitment	Samples from donors with heavy smoking history were excluded.
Ethics oversight	The Institutional Review Board at the University of Chicago has confirmed that because samples used in this study were from deceased donors, they do not qualify as "human subjects".

Note that full information on the approval of the study protocol must also be provided in the manuscript.

Flow Cytometry

Plots

Confirm that:

☒ The axis labels state the marker and fluorochrome used (e.g. CD4-FITC).

☒ The axis scales are clearly visible. Include numbers along axes only for bottom left plot of group (a 'group' is an analysis of identical markers).

☒ All plots are contour plots with outliers or pseudocolor plots.

☒ A numerical value for number of cells or percentage (with statistics) is provided.

Methodology

Sample preparation	Described in methods.
Instrument	FACSAria Fusion; Fortessa X20
Software	Flow cytometry values were recored using Diva software (BD Biosciences). Manual gating for analysis was preformed using FlowJo version 10. Rtsne package for R was used for t-SNE plots.
Cell population abundance	Sort purity was > 98% pure for at least one sample on each day of cell sorting. This value was calculated from a test sample that was sorted and re-run through the cell sorter and is the percentage of leukocyte-gated events that were in the appropriate gate.
Gating strategy	This is shown in Supplementary Figure 1. Based on FSC-A/SSC-A, cells in the lymphocyte gate and singlets according to FSC-W/SSC-W

☒ Tick this box to confirm that a figure exemplifying the gating strategy is provided in the Supplementary Information.
